# Design and Construction of an Azo-Functionalized POP for Reversibly Stimuli-Responsive CO_2_ Adsorption

**DOI:** 10.3390/polym15071709

**Published:** 2023-03-29

**Authors:** Rongrong Yuan, Meiyu Zhang, Hao Sun

**Affiliations:** Department of Materials Science and Engineering, Jilin Jianzhu University, Changchun 130118, China

**Keywords:** porous organic polymer, azobenzene, trans/cis isomerization, CO_2_ sorption

## Abstract

A porous azo-functionalized organic polymer (JJU-2) was designed and prepared via oxidative coupling polymerization promoted by FeCl_3_. JJU-2 exhibited reversibly stimuli-responsive CO_2_ adsorption properties as a result of the trans/cis isomerization of the polymer’s azo-functionalized skeleton. Under UV irradiation and heat treatment, this porous material displayed various porous structures and CO_2_ adsorption properties. The initial Brunauer-Emmett-Teller (BET) surface area of JJU-1 is 888 m^2^ g^−1^. After UV irradiation, the BET surface area decreases to 864 m^2^ g^−1^, along with the decrease of micropores around 0.50 nm and 1.27 nm during the trans-to-cis isomerization process. In addition, CO_2_ sorption isotherms demonstrate an 8%t decrease, and the calculated Qst of CO_2_ has decreased from 29.0 kJ mol^−1^ to 26.5 kJ mol^−1^ due to the trans to cis conversion of the azobenzene side group. It is noteworthy that JJU-2′s CO_2_ uptakes are nearly constant over three cycles of alternating external stimuli. Therefore, this azo-functionalized porous material was a potential carbon capture material that was responsive to stimuli.

## 1. Introduction

Modern industrial-era greenhouse gas emissions have a significant impact on global warming and environmental change. Carbon dioxide (CO_2_) is regarded as one of the most important greenhouse gases, resulting in an increased focus on reducing or capturing CO_2_. Unfortunately, the concentration of CO_2_ in the atmosphere continues to rise globally, endangering human health and the global ecosystem. Consequently, the development of materials and technologies for CO_2_ capture and utilization is crucial for addressing the intractable global problem [1]. Numerous scientists and researchers have focused on the development of functional porous materials to capture and transform CO_2_ under mild conditions [2], including zeolite [3,4], porous carbon [5,6], and metal-organic framework [7,8,9] among others. Porous organic polymers (POPs), as a class of emerging porous materials, are gradually attracting more research efforts due to their excellent physical and chemical stability, large surface area, good designability, and diverse structures, resulting in their wide applications in gas sorption [10,11,12], heterogeneous catalysis [13,14,15], sensor [16,17,18], and drug delivery [19,20,21]. Friedel-Crafts alkylation [22,23], Yamamoto reaction [24,25], Suzuki coupling reaction [26,27], and Sonogashira-Hagihara cross-coupling reaction [28,29] are examples of effective synthetic techniques that have been utilized to create POPs. However, some polymerization techniques have disadvantages that limit their applications in the synthesis of POPs, such as undesirable by-products, high energy consumption, low yield, harsh reaction conditions, and costly catalysts. Among these techniques, oxidative coupling polymerization utilizing FeCl_3_ is regarded as one of the most popular approaches to constructing POF due to its low-cost, large-scale preparation, simple operation, and high universality [30,31,32,33]. On the other hand, carbazole and its derivatives are used as organic monomers to prepare POPs via FeCl_3_-promoted oxidative coupling polymerization due to the large-conjugated system, rigid structure, high reactivity, and excellent photoelectric properties of carbazole and its derivatives. Thus, some carbazole-based POPs have been synthesized and implemented in diverse fields [34,35,36].

In recent years, effective regulation of the interaction between CO_2_ and porous skeletons has become an important area of research, as it is believed to be an energy-saving, accessible, and environmentally-friendly method for capturing and utilizing CO_2_. By altering the skeleton configuration, stimuli-responsive porous materials are an effective way to regulate CO_2_ sorption. Some porous functional materials have response properties to various external stimuli, including pH value [37], temperature [38], and light [39]. Light is a readily available source of renewable energy on Earth, and the introduction of light-responsive groups can alter the structures of porous materials [40]. Recently, metal-organic frameworks (MOFs) as a class of porous organic-inorganic hybrid materials have been utilized in a variety of applications [41,42,43], particularly photoresponsive MOFs for CO_2_ sorption [44,45]. However, MOFs’ poor stability severely restricts their application. POPs are more stable than MOFs and thus provide an excellent platform for light-responsive CO_2_ capture. For high-performance CO_2_ sorption, photoresponsive functional groups are the most important factor in the construction of POPs. Azobenzene is considered one of the readily available and light-sensitive groups. To date, azobenzene-functionalized light-responsive POPs with good photocontrol performance have attracted considerable interest in CO_2_ capture [46,47]. Due to the exceptional photoelectric properties of carbazole and light-responsive azobenzene, carbazole-based POPs with azobenzene fragments serve as a platform for realizing light-responsive CO_2_ sorption.

**Scheme 1:**
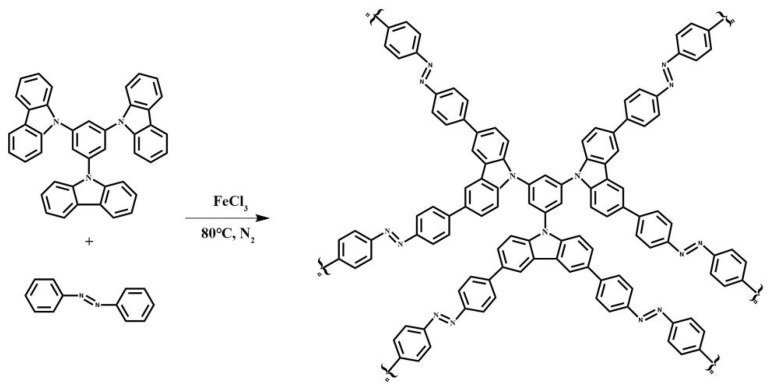
The synthetic process of JJU-2.

Based on our previous reports [48,49], we design and develop a carbazole-based azo-functionalized POP (JJU-2) by coupling 1,3,5-tris(9H-carbazole-9-yl) benzene (TCB) and azobenzene (AB) (Scheme 1). High stability, a large surface area, and well-developed pores characterize the prepared sample. In the meantime, the porous skeleton contains azo functional groups as a light-responsive sensitive component, resulting in stimuli-responsive CO_2_ sorption through cis and trans structure transformations under ultraviolet (UV) irradiation and heat treatments. Furthermore, the controlled CO_2_ sorption/release can be recycled at least three times without difficulty.

## 2. Materials and Methods

### 2.1. Materials and General Methods

Unpurified chemicals were purchased and utilized. UV-Vis spectroscopy was carried out using a Mapada V-1200 (Shanghai Meipuda Instrument Co., Ltd., Shanghai, China). Using ZF-1A ultraviolet analyzer, trans-cis transformations were carried out (JIAPENG, Shanghai, China). IFS 66V/S Fourier transform infrared spectrometer was utilized to collect FT-IR spectra (Bruker Corporation, Rheinstetten, Germany). The ^13^C solid NMR spectrum is performed on a Bruker AV-400-WB instrument (Bruker Corporation, Bill Erica, MA, USA). Rigaku D/MAX 2550 diffractometer records PXRD patterns (Rigaku, Tokyo, Japan) with Cu-Kα (λ = 1.5418 Å) at 50 kV, 200 mA with a 2*θ* range of 4–40° at room temperature. The SEM images were acquired utilizing a MIRA-3 LMU scanning electron microscope (Tescan, Brno, Czech Republic). tecnai G2F20 S-TWIN was used to collect the TEM image (FEI, Hillsboro, WA, USA). The TGA curve was assessed using a PerkinElmer STA6000 thermal analyzer (PERKINELMER, Waltham, MA, USA) in the air. Gas sorptions were performed on Micromeritics before 2020 (Micromeritics Instrument Corporation, Norcross, GA, USA).

### 2.2. Synthesis of JJU-2

In a 100 mL round-bottom flask, 1,3,5-tris(9H-carbazole-9-yl) benzene (229 mg, 0.4 mmol, 1 eq), azobenzene (583 mg, 3.2 mmol, 8 eq), and FeCl_3_ (583 mg, 3.6 mmol, 9 eq) were added, followed by three cycles of vacuuming and inserting with N_2_. Then, 20 mL of dried CHCl_3_ was added to the reaction system using a syringe. The reaction was heated at 80 °C for 24 h in an N_2_ atmosphere. After the mixture cooled to room temperature, it was filtered to obtain the crude product. The product was further washed with HCl (0.3 M) aqueous solution, distilled water, methanol, and dichloromethane in order to remove unreacted monomers and catalyst residues. The product was dried under vacuum for 6 h at 60 °C to produce JJU-2 (orange solid powder, 463 mg, yield of 57%).

## 3. Results

### 3.1. Structural Description

**Figure 1:**
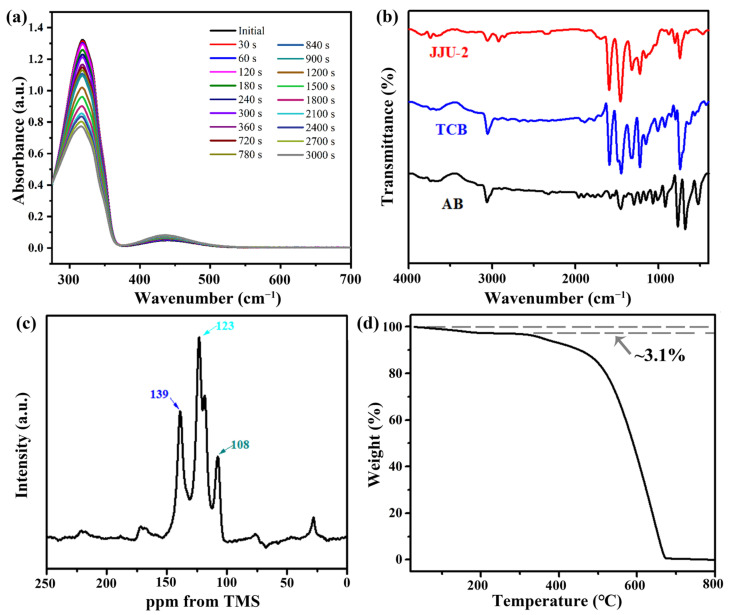
(**a**) UV-Vis adsorption spectra of azobenzene in dichloromethane under the irradiation with 365 nm light. (**b**) FT-IR of monomers and JJU-2; (**c**) ^13^C solid-state NMR (cyan 123, blue 139, green 108); (**d**) TGA of JJU-2.

The UV-visible adsorption spectra of azobenzene in dichloromethane exhibit a typical trans and cis isomerization transformation with obvious π→π* absorption peaks, such as a strong peak at 319 nm and a weak peak at 435 nm (Figure 1a). To examine the successful polymerization of organic monomers and the structure of POPs, Fourier transform infrared (FT-IR) spectra are measured (Figure 1b). By comparing the FT-IR spectra of the initial monomers and JJU-2, the characteristic absorption peaks are summarized as follows: (a) there is an obvious absorption peak at 3000 cm^−1^, which is mainly attributed to the C–H stretching vibration of the hydrogen atom in phenyl ring; (b) the C–H deformation vibration peaks (760–660 cm^–1^) of four adjacent hydrogen atoms on the carbazolyl 1, 2-diXsubstituted phenyl ring is obviously weakened because the number of adjacent hydrogen atoms decreases from four to three; (c) some peaks in the range of 800–690 cm^−1^ belong to the C–H deformation vibration of ring hydrogen atoms of the 1,2-disubstituted phenyl ring of the carbazole group; (d) the peaks around 1300 cm^−1^ corresponds to the –C–N–bond between the aromatic ring and the azobenzene group; (e) FTIR bands located at 1458 cm^−1^ belongs to -N=N- stretching vibration in the azo-functional group in JJU-2, which confirms the successful synthesis of azo-linked POPs [50]. The ^13^C solid-state NMR measurement is then used to examine the structure of the POP of interest (Figure 1c). Due to the presence of aromatic carbon atoms in the benzene ring, the ^13^C solid-state NMR spectrum of JJU-2 exhibits three main peaks between 100 and 150 ppm. Notably, a high peak is observed at 123 ppm, which is attributable to unsubstituted phenyl carbon atoms, whereas two weak signals at 108 and 139 ppm are primarily attributable to substituted phenyl carbon atoms [51]. It further verifies the preparation of the JJU-2 target. As shown in Figure 1d, the thermogravimetric analysis (TGA) curve was measured in air at a heating rate of 10 °C min^−1^. The first weight loss of the as-synthesized JJU-2 is about ~3.1% before ~330 °C, which may be attributed to the adsorbed water and other solvent molecules as the previous similar reports [52,53]. At higher temperatures, the skeleton gradually decomposes because of the oxygen reactions of C, N, and H elements in the air. The TGA curve confirms the good thermal stability of as-synthesized samples.

**Figure 2:**
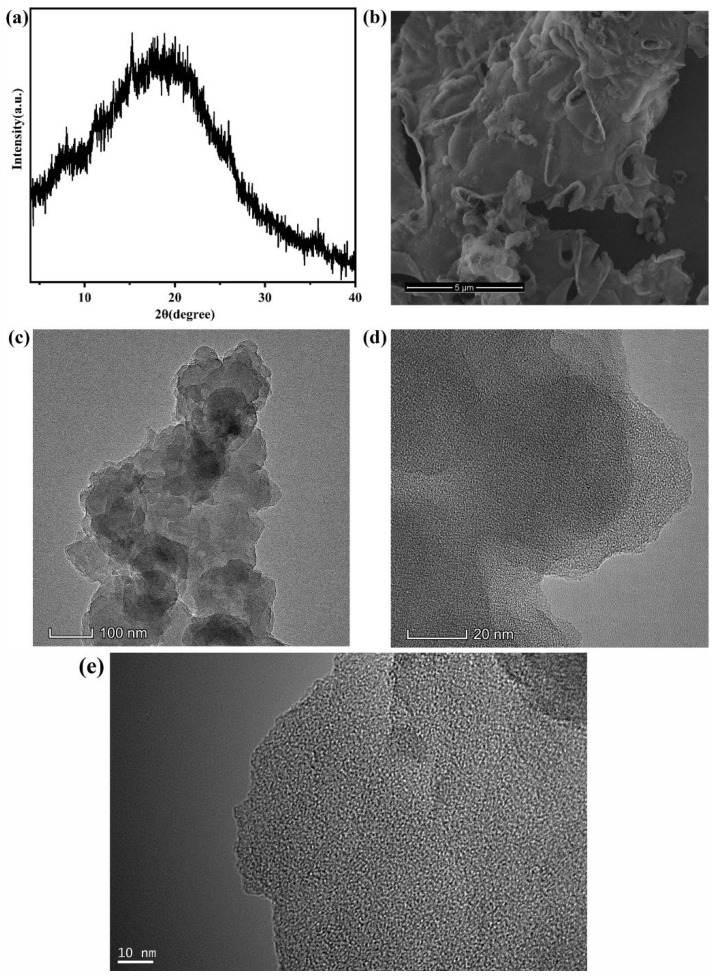
(**a**) PXRD, (**b**) SEM, and (**c**–**e**) TEM images of JJU-2.

In addition, powder X-ray diffraction (PXRD) of as-synthesized POP reveals no diffraction peaks, indicating that as-synthesized JJU-2 is an amorphous solid material (Figure 2a). Using a scanning electron microscope (SEM) and transmission electron microscope (TEM), it is possible to observe the morphology and microstructure of JJU-2. The SEM image reveals that the prepared POP is an irregular crosslinked solid (Figure 2b). Moreover, the TEM images of JJU-2 demonstrate that the synthesized POP has a disordered, worm-like porous structure (Figure 2c–e).

### 3.2. Gas Sorption Properties

**Figure 3:**
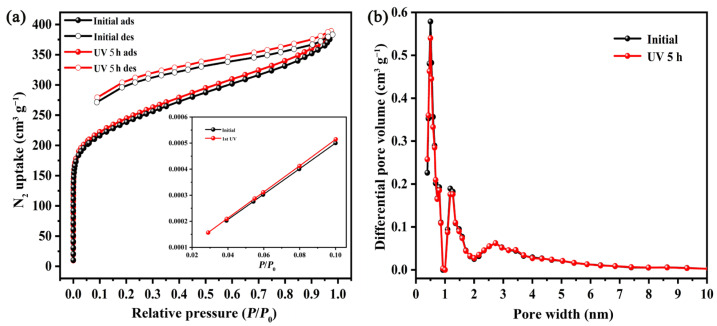
(**a**) N_2_ adsorption and desorption isotherms of JJU-2 with the insert BET surface area plots, and (**b**) pore size distributions of JJU-2. Initial (black) and after UV irradiation for 5 h (red).

In order to examine the internal pores, synthesized JJU-2 is immersed in dichloromethane for two days and then activated at 150 °C for ten hours under a vacuum. The N_2_ sorption isotherms of the initial sample were measured at 77 K, and after 5 h of UV irradiation, they were measured at 77 K once more. As depicted in Figure 3a, the majority of the curves exhibited a type I isotherm with a substantial capillary N_2_ uptake in the low P/P_0_ region. It confirms that JJU-1 is microporous. The desorption curves of both materials showed little hysteresis due to the diffusional limitation of adsorbed N_2_ molecules in restricted micropores [54]. The sample surface areas are computed using the Langmuir and BET models, which are summarized in Table 1. Figure 3a and Table 2 demonstrate BET surface area plots. After 5 h of UV light irradiation, the surface area of the resultant material is smaller than that of the initial material. The NLDFT is utilized to determine the pore size distribution curves for both samples. After UV irradiation, the pore size distributions are between 0.50 nm and 1.27 nm, with a gradual decrease (Figure 3b). Similarly, the pore volumes of materials also undergo a change. Light-responsive azobenzene in the porous skeleton is primarily responsible for the pore change.

**Figure 4:**
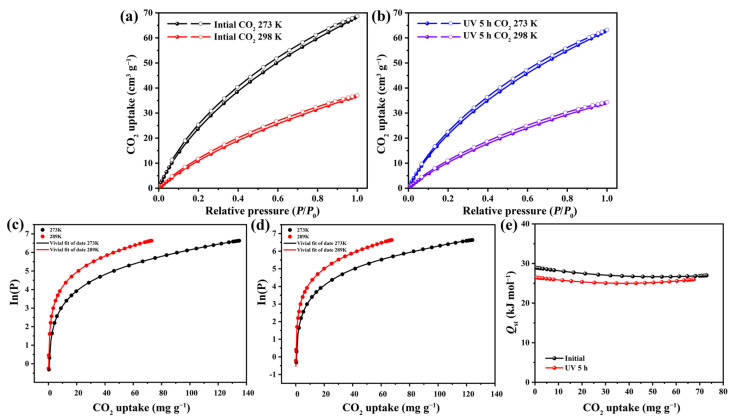
CO_2_ adsorption isotherms of (**a**) initial JJU-2 and (**b**) JJU-2 after UV irradiation for 5 h; the fitting data for calculating the CO_2_ *Q*_st_ value for (**c**) JJU-2-initial and (**d**) JJU-2-1st UV; (**e**) the calculated *Q*_st_ plots.

Based on previous research, the trans/cis isomerization of azobenzene can result in different geometric and dipole variations [55,56,57]. The dynamic structural changes of as-synthesized POP can be used as a light-responsive CO_2_ adsorbent under mild conditions; therefore, the CO_2_ sorption isotherms are carried out at 273 and 298 K, respectively, under 1 atmosphere. The initial POP has a strong capacity for CO_2_ sorption, with a maximum absorption of 68 cm^3^ g^−1^ at 273 K and 37 cm^3^ g^−1^ at 298 K and 1 atm (Figure 4a). Notably, after 5 h of UV irradiation, JJU-2 exhibits different CO_2_ sorption behaviors due to the trans/cis isomerization of azobenzene in its porous structure. As shown in Figure 4b, the CO_2_ sorption amount decreases to 62 cm^3^ g^−1^ at 273 K and 34 cm^3^ g^−1^ at 298 K. Similar to the previous reports [58,59,60], the hysteresis of the CO_2_ desorption is mainly ascribed to the electronic attraction between adsorbed and desorbed CO_2_ molecules, and the interaction between the porous skeleton and CO_2_. Meanwhile, it is probably caused by the pore window and pore expansion in POPs.

Using CO_2_ adsorption at 273 and 298 K, the CO_2_ adsorption enthalpy (Qst) can be calculated to determine the interaction force between CO_2_ and the skeleton of JJU-2 (Figure 4c,d). The calculated CO_2_ *Q*_st_ value of the initial sample is as high as 29.0 kJ mol^−1^, but after 5 h of UV irradiation, the value decreases to 26.5 kJ mol^−1^, proving the trans/cis structure transformation of JJU-2 (Figure 4e). Table 3 provides samples of representative materials [48,49,61,62,63,64,65,66,67,68,69,70].

**Figure 5:**
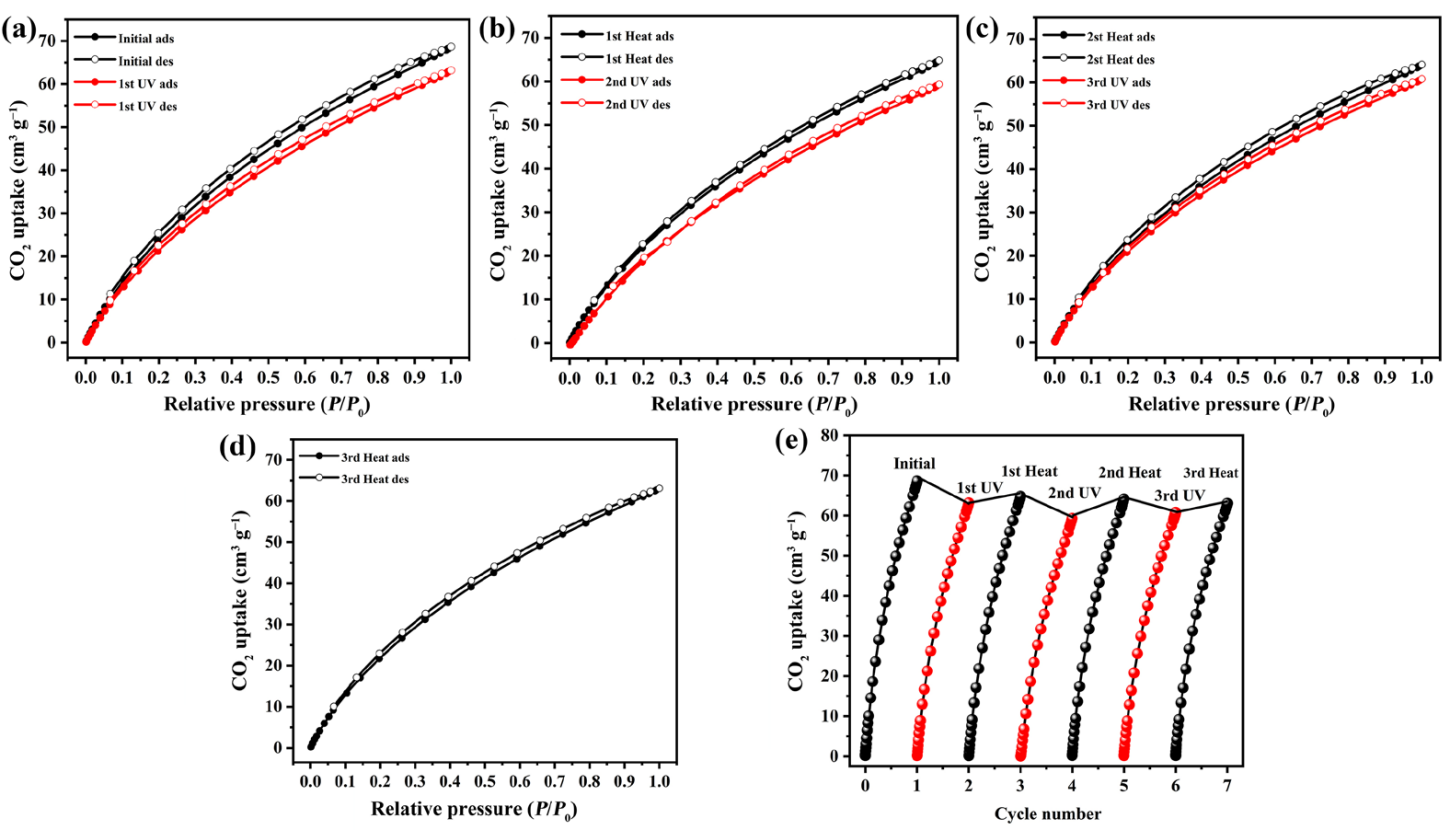
(**a**–**d**) The CO_2_ adsorption isotherms of initial JJU-2 and JJU-2 after UV irradiation for 3 cycles; (**d**) the cyclic responsiveness of JJU-2 after UV irradiation and heat treatments; (**e**) the reversibility of JJU-2 after UV and heat treatment for three cycles.

According to the porous structure of initial and UV-treated samples, the decreased micropore volume may be the main reason for reducing the CO_2_ adsorption capacity of JJU-2 after irradiating by UV light. Reversible circularity is another vital factor for stimulus-responsive CO_2_ sorption porous materials. As illustrated in Figure 5a–e and Table 4, the CO_2_ adsorption isotherms of JJU-2 are repeatedly measured for three cycles after UV irradiation and thermal regeneration, which exhibits that the as-synthesized JJU-2 has good light- and thermal-responsive circularity for adsorbing CO_2_ at 273 K under 1 atm under the controllable external stimulation.

## 4. Conclusions

In conclusion, an Azo-Functionalized POP is designed and synthesized via an oxidative coupling method. The porous structure and light-responsive properties of this POP material are investigated in depth. In POPs, UV irradiation and thermal regeneration can specifically affect the trans/cis transition of azo groups. Under UV irradiation, the capacity for CO_2_ sorption decreases by 8%. In the meantime, this stimuli-responsive POP can revert to its original structure following thermal regeneration. According to recycling tests, the CO_2_ adsorption performance of this synthesized POP can be recycled at least three times via UV irradiation and thermal regeneration. This study aims to provide a method for constructing POPs with CO_2_ adsorption that are reversibly stimulated.

## Figures and Tables

**Table 1:** Gas sorption properties for JJU-2.

POP Materials	SA_BET_^a^ [m^2^ g^−1^]	SA_Langmuir_^b^ [m^2^ g^−1^]	Peak Pore Sizes [nm]	V_Total_^c^ [cm^3^ g^−1^]	CO_2_ Uptake [cm^3^ g^−1^]		*Q*_st_ CO_2_ [kJ mol^−1^]
					273 K	298 K	
Initial	888.1	1002.7	0.27, 1.5	0.60	68.7	37.1	29.0
UV 5 h	864.6	976.3	0.27, 1.5	0.59	63.2	34.4	26.5

Surface areas calculated from BET ^a^ and Langmuir ^b^ models; ^c^ The total pore volume calculated at *P*/*P*_0_ = 0.97.

**Table 2:** BET surface areas of JJU-2.

POP Materials	BET [m^2^ g^−1^]	Slope [g cm^−3^]	*Y*-Intercept [g cm^−3^]	*C*	*Q*_m_ [cm^3^ g^−1^]	Correlation Coefficient
Initial	888.1	0.004890	0.000011	450.072216	204.0409	0.999997
UV 5 h	864.6	0.005023	0.000011	439.286920	198.6445	0.999998

**Table 3:** Summary of CO_2_ uptake and *Q*_st_ value for some reported porous materials.

Materials	BET [m^2^ g^−1^]	V_Total_ [cm^3^ g^−1^]	CO_2_ Uptake (cm^3^ g^−1^) (273 K, 1 Bar)	*Q*_st_ CO_2_ (kJ mol^−1^)	Ref.
JJU-1	467	0.31	45.3	27.1	[48]
JJU-1-UV	469	0.34	40.1	33.1	[48]
POF-Initial	571	0.49	46.2	26.7	[49]
POF-1stUV	549	0.48	41.6	29.7	[49]
Azo-MOP-1	456	0.48	49.6	-	[61]
PAF-36	325	0.25	28.6	27.0	[62]
PAF-36-UV	385	0.27	31.2	28.4	[62]
PAF-37	443	0.27	26.3	36.8	[62]
PAF-37-UV	456	0.28	29.6	40.7	[62]
TAP-1	474	0.74	48.4	35.6	[63]
azo-COP-1	608	0.39	54.7	29.3	[64]
azo-COP-2	703	0.42	56.2	24.8	[64]
Azo-POF-1	712	-	66.7	27.5	[65]
Azo-POF-2	439	-	43.0	26.6	[65]
ALP-4	862	0.50	41.2	28.2	[66]
PCN-250-Fe_3_ (II/III)	1619	-	133.66	-	[67]
PCN-250-Fe_3_ (III)	1598	-	50.81	-	[67]
PCN-250-Al_3_	1874	-	170.48	-	[67]
PCN-250-Sc_3_	1321	-	101.31	-	[67]
PCN-250-In_3_	1224	-	108.15	-	[67]
MS	1015	0.96	-	-	[68]
DAL(1)@MS	627	0.46	-	-	[68]
DAL(2)@MS	589	0.40	50	-	[68]
DAL(2)@MS UV	-	-	33	-	[68]
DAL(3)@MS	512	0.31	-	-	[68]
BCzMB@PON	865	-	90.048	31.96	[69]
TPA@PON	829	-	86.24	37.98	[69]
CZ@PON	592	-	96.544	42.01	[69]
HMC-1	855	0.2968	235.2 (273 K, 3 bar)	-	[70]
HMC-2	425	0.1920	306.88 (273 K, 3 bar)	-	[70]
HMC-3	526	0.1618	318.08 (273 K, 3 bar)	-	[70]
JJU-2	888.1	0.60	68.7	29.0	This Work
JJU-2-UV	864.6	0.59	63.2	26.5	This Work

**Table 4:** The CO_2_ maximum adsorption amounts for JJU-2 at 273 K and 1 bar.

JJU-2	CO_2_ Uptake [cm^3^ g^−1^]
Initial	68.7
1st UV	63.2
1st Heat	64.8
2nd UV	59.3
2nd Heat	64.1
3rd UV	60.8
3rd Heat	63.1

## Data Availability

No new data were created or analyzed in this study. Data sharing is not applicable to this article.
